# Multivariate classification techniques and mass spectrometry as a tool in the screening of patients with fibromyalgia

**DOI:** 10.1038/s41598-021-02141-1

**Published:** 2021-11-19

**Authors:** Marcelo V. S. Alves, Lanaia I. L. Maciel, Ruver R. F. Ramalho, Leomir A. S. Lima, Boniek G. Vaz, Camilo L. M. Morais, João O. S. Passos, Rodrigo Pegado, Kássio M. G. Lima

**Affiliations:** 1 Institute of Chemistry, Biological Chemistry and Chemometrics, Federal University of Rio Grande do Norte, Natal, 59072-970 Brazil; 2 Institute of Chemistry, Federal University of Goiás, Samambaia St., Goiânia, GO 74690-900 Brazil; 3 Estácio de Sá Goiás, North Regional, Goiânia, GO 74063-010 Brazil; 4 School of Pharmacy and Biomedical Sciences, University of Central Lancashire, Preston, PR1 2HE UK; 5 Postgraduation Program in Rehabilitation Sciences, Faculty of Health Science of Trairí, Federal University of Rio Grande do Norte, Trairí St., Santa Cruz, RN 59200-000 Brazil

**Keywords:** Mass spectrometry, Computational models, Machine learning

## Abstract

Fibromyalgia is a rheumatological disorder that causes chronic pain and other symptomatic conditions such as depression and anxiety. Despite its relevance, the disease still presents a complex diagnosis where the doctor needs to have a correct clinical interpretation of the symptoms. In this context, it is valid to study tools that assist in the screening of this disease, using chemical work techniques such as mass spectroscopy. In this study, an analytical method is proposed to detect individuals with fibromyalgia (n = 20, 10 control samples and 10 samples with fibromyalgia) from blood plasma samples analyzed by mass spectrometry with paper spray ionization and subsequent multivariate classification of the spectral data (unsupervised and supervised), in addition to the treatment of selected variables with possible associations with metabolomics. Exploratory analysis with principal component analysis (PCA) and supervised analysis with successive projections algorithm with linear discriminant analysis (SPA-LDA) showed satisfactory results with 100% accuracy for sample prediction in both groups. This demonstrates that this combination of techniques can be used as a simple, reliable and fast tool in the development of clinical diagnosis of Fibromyalgia.

## Introduction

Fibromyalgia (FM) is a rheumatologic disorder that causes chronic pain and other symptomatic conditions such as depression, anxiety and sleep and memory disorders^1,2^. This disorder is common in clinical rheumatology routine, with a worldwide prevalence between 0.5 and 5%, affecting women approximately seven times more than men^3^. Despite its relevance, the disease still has a complex diagnosis where the physician needs to have a correct clinical interpretation of the symptoms, since they are similar to other rheumatologic diseases^3,4^.

The correct diagnosis of this disorder requires studies on tools that help in the screening of individuals affected with this disease. One of the techniques used for this purpose is Mass Spectrometry (MS) coupled with Liquid Chromatography techniques (LC–MS)^5^ e Gas Chromatography (GC–MS)^6^. These techniques have a high analytical performance, but they require laborious and extensive sample preparation procedures, high consumption of solvents and reagents, and generate chemical residues^7^. Thus, it is necessary to develop analytical methods that prioritize the resolution of these problems. With Ambient Mass Spectrometry (AMS)^8^, and the introduction of techniques such as: direct Analysis in Real Time (DART) and Desorption Electrospray Ionization (DESI), there is a new way to generate ions in MS^7,9^. AMS is characterized by ion generation under ambient conditions and direct compound analysis in its native environment with minimal or no sample preparation^10^.

Among the various techniques belonging to this group, there is the speed and versatility of Paper Spray Ionization (PSI)^11^. In PSI, the sample (liquid^12^ or solid^13^) is deposited on the surface of a triangular paper followed by the application of a high voltage and solvent, forming a spray on the tip of this paper containing sample droplets that are sent to the MS^14^. This method facilitates the analysis of biofluids quickly and quantitatively^15^. PSI is applicable to a wide range of biological compounds, including peptides, small nucleotides, phospholipids and other organic compounds^16^, being used in the quantification of drugs in the plasma of patients^17^ and in clinical studies predicting some types of cancer^18,19^. Considering that there is no standard instrumental technique for fibromyalgia, the diagnosis is clinical by elimination. The PSI-MS technique was chosen because it is innovative, does not require sample preparation and has a very relevant analytical power, lowering analysis costs when compared to ESI–MS, for example, and can be implemented in hospital routines.

In view of the versatility of the PSI method and in order to better understand the resulting results, it is worth involving an analysis of the metabolites so that it is possible to identify and quantify the main metabolites of the biological system^20^. Metabolomic analysis is typically characterized in two approaches^21^: targeted approach on selected metabolite or metabolite class with known chemical properties; and untargeted approach, measuring all possible metabolites providing hypotheses for further testing. The study of metabolomics contributes to a better understanding of the biological characteristics of different types of cancer^22^, as well as in understanding the association with indicator biomolecules for the diagnosis of fibromyalgia^23^. To make this possible, multivariate statistical analysis is commonly used^24^, where the combinatorial effect of multiple variables is considered and can be characterized in unsupervised techniques, which refer to methods that identify hidden structures in the data without knowing the class labels, and supervised techniques that use the information from the class label to build the classification model^21^.

Several types of chemometric algorithms are reported for pattern recognition and classification of MS data, in particular to discriminate between healthy and cancer samples^24^. Some reports on the investigation of biological and metabolomic markers in biofluids from patients with FM are also located in the literature^23,25,26^, but without the occurrence of application of chemometric tools for group classification. In a recent study^2^ the blood plasma of control and fibromyalgia patients is used, in which it was possible to reach good results of discrimination of these groups from infrared (IR) spectroscopic analysis coupled with chemometric techniques. In this case, despite the good results in IR, the spectra obtained are difficult to interpret and have low resolution for elucidating molecules of clinical interest, and consequent detection of FM in blood smear. However, working with IR provides a good suggestion for using classification tools, seeking to optimize the best performance results.

In this study, nine algorithm combinations were used (PCA-LDA, PCA-QDA, PCA-SVM, SPA-LDA, SPA-QDA, SPA-SVM, GA-LDA, GA-QDA and GA-SVM) that are executed and compared to discriminate between blood plasma samples from control and fibromyalgia patients. These algorithms used the spectra obtained through the PSI-MS technique, providing a practical and reliable method without complex sample preparation. In addition, associations of selected variables with already cataloged metabolites are also explored, indicating the possible presence of biological fingerprints in the study groups.

## Results

**Figure 1:**
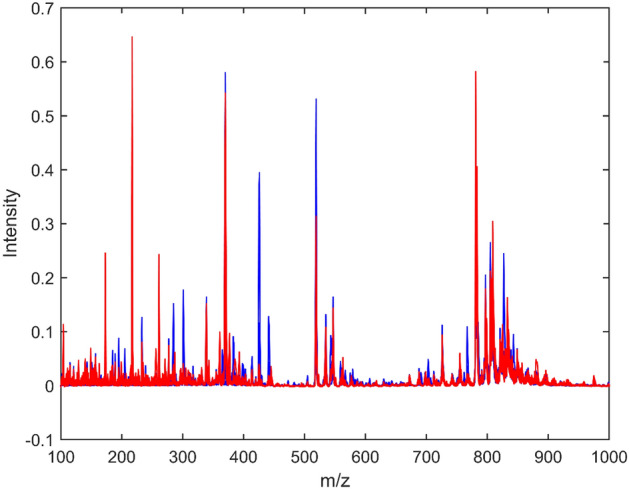
Baseline corrected mass spectra for the control group (blue) and FM group (red) obtained by PSI(-)-MS.

It is possible to visualize in Fig. 1 the overlap along the MS signal that does not allows visual differentiation between the control group (CG) and the fibromyalgia group (FG). The raw spectra are pre-processed with weighted automatic least squares baseline correction and vector normalization^27^. Spectral samples were divided into training (70%) and test (30%) groups using the Kennard-Stone sample selection algorithm. In this division, the set of training samples is used in the construction of the model, while the set of test samples are used in the prediction of model performance. The spectra resulting from the pre-processing are submitted to classification of FG and CG groups. Classification was performed with algorithm combinations like Principal Component Analysis (PCA), Successive Projection Algorithm (SPA) and Genetic Algorithm (GA) combined with supervised algorithm like Linear Discriminant Analysis (LDA), Quadratic Discriminant Analysis (QDA); and, Support Vector Machines (SVM); proposing PCA-LDA, PCA-QDA, PCA-SVM, SPA-LDA, SPA-QDA, SPA-SVM, GA-LDA, GA-QDA and GA-SVM models to optimize the results and identify those with the best performances.

**Figure 2:**
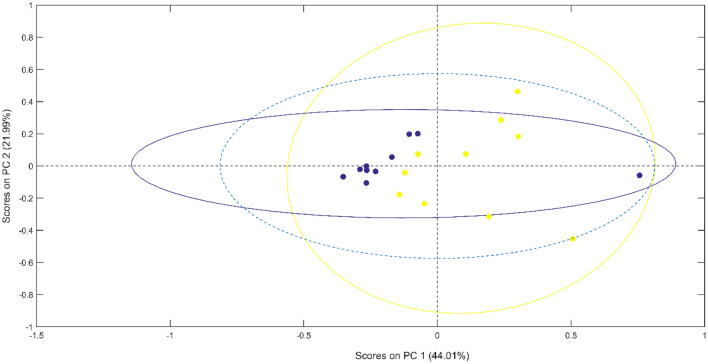
PCA scores on PC1 versus PC2 for samples from the control group and the FM group, with confidence ellipse in each group. The percentage of total variance is described for each PC (in parentheses). The blue dotted circle represents a 95% confidence level.

To start distinguishing between classes, pattern recognition algorithms were used. At first, the PCA is used as an exploratory analysis for the dataset, where the score plots with confidence ellipse are shown in Fig. 2. The plot of scores of the first two principal components (PC1 and PC2) demonstrate a good separation of the two classes, with the samples from the control group clustered in a region different to the samples from the case group. The presence of a sample from the control group displaced to the right of the others is also visible, which may indicate an outlier and the presence of a patient with FM in the control group. For this initial analysis, sensitivity and specificity calculations were made with 100% accuracy in discriminating groups. These results suggest the PCA as a good tool for classification, demonstrated in the pattern of discrimination observed in the score plots.

Despite the good performance of the exploratory analysis, it is possible to observe that the confidence ellipses demonstrate a large region of intersection between groups, which proposes a more elaborate exploratory method. We opted for subsequent supervised analyzes, also considering the limited number of samples and confirmation of the results. In the further investigation of the data, supervised discriminant analyzes (LDA and QDA) associated with PCA data reduction were used. In this case, the first 5 principal components (PC) were applied with an accumulated explained variance of 76.33%. For these models, the results are more expressive for linear classification techniques, with 100% sensitivity and specificity for the control group, while for the group of people with FM the values decrease to 33.3% (Table 1). By associating variable selection techniques with discriminant analyzes in SPA-LDA, SPA-QDA, GA-LDA and GA-QDA, the best results are observed for the supervised methods obtained in this study, in which the SPA-LDA model presented 100% accuracy in both classes and the GA-LDA model with 95.8% accuracy for the CG and 73.6% for the FG. The low results in the other models, especially in the QDA combinations, may imply a bad adjustment due to the absence of large differences in the variance structures between the classes^28^. For the mentioned LDA models, as well as the good classification results, the possibility of exploring the most significant variables in the distinction of the GC and GF groups can also be demonstrated, which comprise the investigation of possible relationships with the metabolomics present in the biological samples of individuals participants from each class (Table 2).

**Table 1:** Sensitivity and specificity results, in percentage, for the control group and with FM, demonstrating the effectiveness in classifying the groups using the supervised models.

Model	Control group		Fibromyalgia group	
	Sensitivity (%)	Specificity (%)	Sensitivity (%)	Specificity (%)
PCA-LDA	100	100	33.3	33.3
SPA-LDA	100	100	100	100
GA-LDA	100	91.7	69.4	77.8
PCA-QDA	50	0	0	0
SPA-QDA	100	100	33.3	33.3
GA-QDA	35	0	43.3	0
PCA-SVM	100	100	66.7	66.7
SPA-SVM	66.7	66.7	100	100
GA-SVM	38.9	38.9	100	87.5

**Table 2:** Selected variables (m/z) in PCA and supervised models.

MODEL	Table with selected variables (m/z)									
PCA (PC1)	173	217	261	339	370	519	781			
PCA (PC2)	173	217	261	370						
SPA-LDA	1	118								
GA-LDA	55	360	376	475	592					
	10	44	475	544	602	638	658			
	64	525	649	651	791	832				
SPA-QDA	77	118								
GA-QDA	65	135	202	307	450	528	745	823	887	898
	108	330	365	493	520	605	693	751	777	847
	19	76	163	231	322	360	374	487	589	833
SPA-SVM	77	118								
GA-SVM	65	77	80	95	167	194	390	590	593	698
	3	112	138	173	280	348	482	610	833	878
	77	218	296	390	457	646				

Another supervised technique of nonlinear classification, in addition to QDA, used in this paper in search of best results, was the SVM using the Polynomial Base Function (PBF) kernel for the spectral dataset, as indicated in a study with blood samples^29^. For the PCA-SVM, good performance was presented for the CG group with 100% accuracy (Table 1). With the SPA-SVM and GA-SVM variable selection algorithms, the results also indicate good distinctions between CG and FG, with emphasis on the classification of the group with FM in the SPA-SVM model with 100% accuracy in this group, and the GA-SVM where 100% sensitivity and 91.66% specificity were achieved for individuals with the disease. Associations with metabolomics were also raised for the small set of selected variables (Table 2).

**Figure 3:**
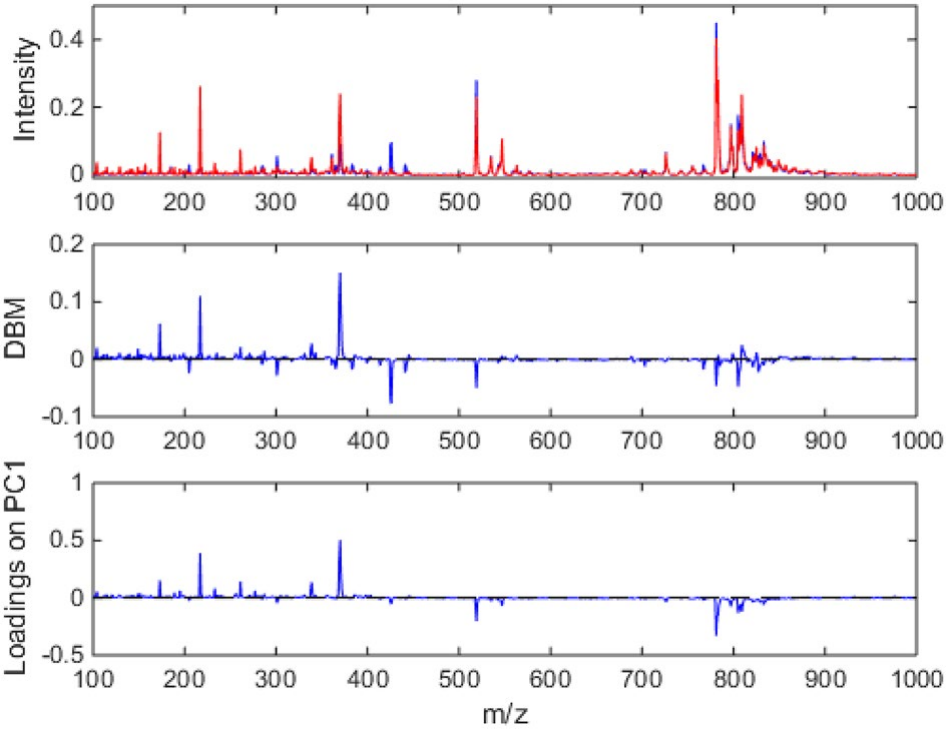
The spectral mean with all samples, the difference between the spectral means (DBM) and the chemical profile of the samples for PC1 are shown.

In the supervised models with variable selection, for a universe with more than 900 variables present in the MS dataset, a total of sixty-six values were collected due to the need for three executions of the GA models, because it is a non-deterministic algorithm. Thus, among the variables represented in more than one model there are: 65, 77, 118, 173, 360, 390 and 475 m/z. Among these m/z values, the 118 m/z is present in three different SPA models. These selected variables and for those indicated in the differences between the class means and chemical profile of the samples in the PCA (Fig. 3) forward to the study of metabolites.

Platforms with information in the human metabolomics database such as HMDB^30^, LIPID MAPS^31^ and PubChem^32^ were used. Among the compounds identified from the m/z values selected in all worked models, the following classes of compounds are observed: organics acids and derivatives, inorganic compound, nucleotides, benzenoids, organohalogen and organosulfur. For the value 118 m/z the database directed to compounds organoheterocyclics.

The observations were carried out on the HMDB and LIPID MAPS platforms following the positive mode ion settings in M + H and molecular mass tolerance in ± 0.01 m/z. Among the identified metabolites, those with the highest value attributed are Lysophosphatidylcholine (LysoPC) found in the HMDB library as LysoPC (16:1/0:0) and LysoPC (18:2/0:0) compounds. In the LIPID MAPS platform there was no association with any information from the database about these compounds. The relationship with the compound LysoPC (16:4) was indentified on the PubChem platform and the other metabolites of the HMDB library were confirmed.

## Discussion

Studies carried out by the American College of Rheumatology offer good results for differentiating between groups of patients with and without fibromyalgia, taking into account the occurrence of 11 of the 18 sensitive pain points for FM symptoms^33^, the accuracy obtained in this classification was 84.9% with a sensitivity of 88.4%. In a more recent study using blood plasma samples from groups with and without FM and readings in mid-infrared spectroscopy coupled with multivariate classification^2^, the accuracy results reached values of 84.2% and sensitivity of 89.5% using the GA-LDA model. For this paper, using paper spray ionization mass spectrometry, the best results provided 100% accuracy for SPA-LDA, in addition to classification with PCA that also recognized all case group (FG) samples as patients with fibromyalgia. When comparing these results it is possible to observe good values for distinction between the classes, but the emphasis is on the SPA-LDA model through the PSI-MS analysis technique, which indicates the good potential of this methodology as a support in the screening for the disease.

Thus, the use of different classification methods aims to expand the possibilities of satisfactory results. In the case of unsupervised analysis with PCA, in addition to demonstrating good ability to separate the groups, the use also aimed to reduce multivariate data in order to match the complexity of the subsequent supervised classifiers, with quantities of data available to avoid overfitting or under-training^34^. The good results with PCA can be related to the reduced number of samples, which could also to indicate the impossibility of using supervised techniques. However, this study explores the potential of MS for the smallest number of samples, considering that MS has high analytical sensitivity for small amounts of available biological fluids, demonstrating high resolution with accurate mass data for structural elucidation^35^. The SPA and GA algorithms, through the calculations performed to classify the groups, also indicate selected variables that may be associated with the presence of FM in the case group. The LDA supervised models are recurrent in several clinical studies of this nature, such as in cancer^36,37^ and viral diseases^38–40^, because they work with processes with less complex characteristics. It is also common in the literature to find similar studies that expand the classifications to more robust models such as QDA^41,42^ and SVM^43^, other researches seek to further expand the treatment of spectral samples, submitting them to these three types of techniques (LDA, QDA and SVM) and comparing the better accuracy results given the complexities of multivariate data^2,24^. This last approach is worked on and observed in this article.

This work also explored the possibility of associating variables selected by the models used with metabolomics cataloged in the literature in order to indicate possible biochemical markers in the identification of patients with FM. Among sixty-six selected variables, from a universe with more than 900 variables representing the range of mass/charge ratios analyzed, eight m/z ratio values are recurrent in more than one model while the 118 m/z variable is suggested by all SPA models. Despite not identifying new metabolites present in the database, the observation of greatest interest is to confirm the presence of Lysophosphatidylcholine (LysoPC) compounds^30^ suggested by a study of patients with FM indicating a possible role of LysoPC as a biomarker, with results indicating an increase in this metabolite in patients with Fibromyalgia^44^.

The exploratory analysis with PCA and the supervised analysis with SPA-LDA demonstrated satisfactory results with 100% accuracy for predicting samples in both groups (CG and FG) using the PSI-MS technique. Other models had more reasonable values such as GA-LDA, PCA-SVM and SPA-SVM, which may indicate overtraining for the SPA-LDA model, but all classification techniques were submitted to confounding tests that validate the effectiveness of the tool, which it consists of new processing with the mutual exchange between the CG and FG groups to try to deceive the mathematical model. The representation of variables selected by the models also indicated possible lines of investigation for the understanding of biological fingerprints in the identification of patients with FM, despite the absence of new metabolites associated with these variables in the referenced database, based on blood samples, it was possible to confirm the presence of Lysophosphatidylcholine that represent compounds associated with the disease, according to the literature. Regarding the results found in the classification analyses, the study proposes the best results for two simple and easy to perform algorithms, when compared to the others. The results of this study also surpass the reference methods already used, demonstrating another method with good efficacy in FM screening. Despite the optimistic results, it is worth remembering that the sample universe was reduced when compared to the reference methods. Another important consideration is the size of the blood sample collected, as only plasma is used in the instrumental analysis, keeping the main biochemical information, which may indicate that statistics are kept for larger samples.

Thus, considering that the experimental technique with PSI-MS and spectral analysis by using PCA as an unsupervised technique and SPA-LDA as a supervised technique reach 100% sensitivity and specificity for both classes of individuals with FM and without the disease, this has promising potential as an analytical approach with good clinical outcomes. This combination of techniques can be used as a simple, reliable and fast tool in making clinical diagnosis of Fibromyalgia. In addition to these considerations, the detection of metabolomics is also a positive factor in the study, proposing the association of this information with possible biological markers for the disease.

## Methods

### Sample

This case–control study was performed following the ethics standards of the Declaration of Helsinki and was approved by the local institutional ethics committee at the Onofre Lopes University Hospital (Federal University of Rio Grande do Norte, Natal, Brazil) under registration number 2.631.168. Informed consents were obtained from all subjects of this study; and all experimental protocols complied with the ethics guidelines. For this study, 20 plasma samples were selected, 10 samples from patients in the control group and 10 samples from patients with FM. Data were collected from July 2018 to March 2019 and recruitment was carried out throughout this period. The study was carried out at the Clinical Epidemiological Laboratory of the Federal University of Rio Grande do Norte, Natal (RN), Brazil. Sociodemographic data (gender, age, education, occupation, marital status and ethnicity), clinical data (impact of fibromyalgia, anxiety, pain and quality of life) and 10 mL of blood were collected from each patient on the same day. In the Supplementary Information, further conditions for sample collections are described (S1).

### Conventional PSI-MS

For each selected plasma sample, a small 0.05 mL aliquot was removed and applied to triangular paper (Whatman grade 1, GE healthcare, USA, with 1.5 cm sides) left at room temperature (25 °C) until drying. Triangular papers containing small aliquots of blood were positioned in front of the mass spectrometer (at a distance of 4 mm between the tip of the paper and the entrance of the mass spectrometer). The dry paper was held by a metal clip connected to the voltage source of the mass spectrometer, with the tip of the paper at a distance of approximately 5 mm. 10 µL of methanol (0.1% formic acid v/v) was applied to the paper to form the electrospray for MS analysis. Analyzes were performed in triplicate measures.

### Instrumental parameters

Mass spectra were obtained using a Thermo Scientific LTQ-XL Linear Ion Trap spectrometer. The optimized parameters were as follows: negative ionization mode; capillary temperature 275 °C; 15 V capillary voltage; 4 kV spray voltage; tube lens 50 V. Mass spectra were acquired using Thermo Tune plus software and processed for chemometric analyzes using Xcalibur Analysis package software (version 2.0, Service release 2, Thermo Electron Corporation).

### Computational analysis

Spectral data were processed using MATLAB R2014b software (MathWorks Inc., Natick, USA) with PLS Toolbox version 7.8 (Eigenvector Research Inc., Wenatchee, USA). The set of spectral samples was subjected to pre-processing with weighted automatic least squares baseline correction and vector normalization. Spectral samples were divided into training (70%) and test (30%) groups using the Kennard-Stone sample selection algorithm. In this division, the set of training samples is used in building the model, while the set of testing samples are used in predicting the model's performance. The description of the classification methods used in this study can be found in the Supplementary Information (S2).

## Supplementary Information

Supplementary Information.
