# Supplementary material for: Multivariate classification techniques and mass spectrometry as a tool in the screening of patients with fibromyalgia
Source: Sci Rep. 2021 Nov 19;11:22625. doi: 10.1038/s41598-021-02141-1 (PMC8604931; doi:10.1038/s41598-021-02141-1)
Supplement: Supplementary file 1 — Supplementary Information. [file 41598_2021_2141_MOESM1_ESM.docx]

Multivariate classification techniques and mass spectrometry as a tool in the screening of patients with fibromyalgia

Marcelo V. S. Alves^1^, Lanaia I. L. Maciel^2^, Ruver R. F. Ramalho^2^, Leomir A. S. Lima^3^, Boniek G. Vaz^2^, Camilo L. M. Morais^4^, João O. S. Passos^5^, Rodrigo Pegado^5^, Kássio M. G. Lima^1*^

**Supplementary Information**

**S1** **(Sample):** This case-control study was carried out following the ethical standards of the Declaration of Helsinki and was approved by the local institutional ethics committee of the Onofre Lopes University Hospital (Federal University of Rio Grande do Norte, Natal, Brazil) under registration number 2,631.168. Informed consent was obtained from all subjects in this study; and all experimental protocols followed ethical guidelines. The subjects were recruited from social networks and from the medical clinic of Hospital Universitário Onofre Lopes (HUOL). The following inclusion criteria were adopted: (a) medical diagnosis of fibromyalgia according to the ACR / 2010; (b) ability to answer the questionnaire and understand the purpose of this study; (c) patients not undergoing physical therapy or rehabilitation programs in the previous three months; and (d) age ranging from 18 to 80 years. The exclusion criteria were: (a) physical and/or organic problems, when these compromised the application of the questionnaire; and, (b) rheumatic and/or autoimmune diseases including chronic fatigue syndrome, rheumatoid arthritis, gout and lupus.

**S2 (Computational Analysis):** Principal Component Analysis (PCA) is a powerful and versatile tool capable of providing an overview of complex multivariate data^33^. Using PCA it is possible to reduce a large volume of MS data into some principal components (PC) representing most of the original information^24^. In Successive Projections Algorithm (SPA), the projection operations are used to choose subsets of variables with a small degree of multicollinearity, allowing the detection of specific spectral bands^34^. The Genetic Algorithm (GA) draws inspiration from natural evolution to become a robust and efficient algorithm at the same time^35^, providing selected variables at each execution of the model, which may indicate a good strategy for exploring the features present in the samples analyzed by MS. Both PCA as an unsupervised classification method and SPA and GA as variable selection algorithms can be used in conjunction with other supervised techniques such as: Linear Discriminant Analysis (LDA) and Quadratic Discriminate Analysis (QDA) that aim to find limits that separate groups or samples, with LDA obtaining linear limits where a straight line divides the variable space into regions, and QDA obtaining quadratic limits where a quadratic curve divides the variable space^36^. These discriminant analysis techniques, combined with data reduction techniques, are used in several studies involving discrimination of spectral data from biological samples, in particular for cancer studies, and to a lesser extent the Support Vector Machines (SVM) algorithm is used considering the multidimensionality of data and non-linear limits^37^.

The PCA application proposes the reduction of spectral data into principal components (PCs) orthogonal to each other. These components are composed of scores and loadings, where it is possible to observe the disposition of the samples of the case and control groups. The other chemometric models rely on algorithms such as PCA, SPA and GA combined with LDA, QDA and SVM. In LDA and QDA, the processing is based on the calculation of the Mahalanobis distance between the samples, in which the LDA classification ($L_{ik})$ and QDA ${(Q}_{ik})$ follow the calculations^24^:

$L_{ik}=(x_{i}- \bar{x}_{k})^{T} C_{pooled}^{-1}\left( x_{i}- \bar{x}_{k} \right)-2log_{e}\pi_{k}$ (1)

$Q_{ik}=(x_{i}- \bar{x}_{k})^{T} C_{k}^{-1}\left( x_{i}- \bar{x}_{k} \right)+log_{e}\left| C_{k} \right|-2log_{e}\pi_{k}$ (2)

For this calculation $x_{i}$ refers to the vector that contains the input variables for a sample *i****;*** $\bar{x}_{k}$ is the mean vector of the class *k;* $C_{pooled}$ is the pooled covariance matrix; while$C_{k}$is the variance-covariance matrix of the class *k*; and,$\pi_{k}$ is the prior probability of the class *k*.

For SVM, the calculation follows the formula^34^:

$\hat{y}(x)= sign \left( \sum_{i=1}^{N_{SV}} \alpha_{í}y_{i}K\left( x_{i}, z_{i} \right)+b \right)$ (3)

$K\left( x_{i}, z_{i} \right)$ is the Kernel function for $x_{i}$ e $z_{j}$ which are input variables for different class. $\alpha_{í}$ is the Lagrange multiplier; $y_{i}$ is the number of training class members; and *b* is the polarization parameter. $\hat{y}(x)$ represents the final class predicted for spectrum *x*.

The classification performance of the algorithms using in this study is evaluated using sensitivity (eq.4), specificity (eq.5) and accuracy (eq.6), using test set of samples for each group (case and control).

$SENS=\left( \frac{TP}{TP+FN} \right) x 100$ (4)

$SPEC=\left( \frac{TN}{TN+FP} \right) x 100$ (5)

$AC=\left( \frac{TP+TN}{TP+FP+TN+FN} \right) x 100$ (6)

where TP stands for true positive, FP for false positive, TN for true negative and FN for false negative.
